# Seric concentrations of copper, chromium, manganesum, nickel and selenium in aerobic, anaerobic and mixed professional sportsmen

**DOI:** 10.1186/s12970-018-0212-4

**Published:** 2018-02-13

**Authors:** Marcos Maynar, Francisco Llerena, Ignacio Bartolomé, Javier Alves, María-Concepción Robles, Francisco-Javier Grijota, Diego Muñoz

**Affiliations:** 1 Department of Physiology, School of Sport Sciences, University of Extremadura, University Avenue, 10003 Cáceres, Spain; 2 Department of Medical-Surgical Therapeutics, School of Medicine, University of Extremadura, Elvas Avenue, 06071 Badajoz, Spain; 3 Department of Sport Sciences, School of Sport Sciences, Pontifical University of Salamanca, Henry Collet Street, 53, 37007 Salamanca, Spain; 4 Department of Physical Education and Sport, School of Sport Sciences, University of Extremadura, University Avenue, 10003 Cáceres, Spain

**Keywords:** Essential metals, Serum, Metabolism, Exercise, Adaptations

## Abstract

**Background:**

The aim of the present study was to determine changes in serum concentrations of trace elements Cooper (Cu), Chromiun (Cr), Manganesum (Mn), Nickel (Ni) and Selenium (Se) in high-level sportsmen.

**Methods:**

Eighty professional athletes of different metabolic modalities, were recruited before the start of their training period. Thirty one sedentary participants of the same geographic area constituted the control group. Cu, Cr, Mn, Ni and Se analysis was performed by Inductively Coupled Plasma Mass Spectrometry (ICP-MS).

**Results:**

Higher concentrations of Cr (*p* < 0.001), Mn (*p* < 0.085), and Ni (*p* < 0.001) were found in sportsmen in comparison to controls, inversely, Se values were lower (*p* < 0.001) among sportsmen. When sportsmen were classified by metabolic modalities, it was found that aerobic-anaerobic group had higher (*p* < 0.01) Cu concentrations than controls and the other sportsmen. The highest Cr values were found in aerobic participants. For Mn, the major levels were found in aerobic and aerobic-anaerobic groups as well (*p* < 0.001). The lowest Se levels were found among anaerobic sportsmen (*p* < 0.001).

**Conclusion:**

This research showed that daily, continuum physical training induced alterations in serum essential minerals concentrations, as well as that these changes can be dependent of the exercise modality practiced.

## Background

Essential trace elements such as chromium (Cr), copper (Cu), manganese (Mn), nickel (Ni) and selenium (Se) are needed in small amounts in the human body. Despite of the minuscule organic quantities, all of them are necessary for the maintenance and regulation of many biological functions. Cr is linked to the carbohydrates metabolism, modifying blood glucose levels and acting as an insulin enhancer. Besides, Cr is also involved in the lipids metabolism and in the protein anabolism [1–3]. Ni takes part in the tissular regulation of phospholipids, triglycerides, urea, glucose, glycogen and ATP levels [4, 5]. Cu, Mn and Se are critical elements of the three main cellular antioxidant enzymes, the copper-zinc-superoxide dismutase (Cu-Zn-SOD), the manganese-superoxide dismutase (Mn-SOD) and the glutathione-peroxidase (GSH-Px), respectively. It has been demonstrated that all three enzymes suffer exercise-induced adaptations [6]. Furthermore, lower concentrations of Cu, Mn and Se could induce injuries in body functions and physical performance downgrade [7]. It is known that physical activity leads to many metabolic changes in human organism; intense training exercises may increase essential trace elements requirements, either by augmented degradation rates or by increased losses from the body. However, the differences in organic concentrations of these elements of sportsmen in comparison to sedentary population have been poorly studied. Furthermore, it has been recently reported that physical training may induce changes in serum concentrations of several biological minerals [8].

The aim of this study was to determine differences in serum Cr, Cu, Mn, Ni and Se values in sportsmen in comparison to sedentary population as well as to evaluate if different metabolic modalities induced differences in these serum minerals concentrations.

## Methods

### Participants

This research was carried out under the Helsinki Declaration ethic guidelines, updated at the World Medical Assembly in Seoul in 2008, for research with human participants. All the participants were previously informed about the purpose of the study and gave their voluntary signed informed consent.

Eighty male professional athletes and 31 sedentary males participated in the present survey. All of them were living in Cáceres (Spain) and had been living in the same region for, at least, two years. The athletes had trained regularly for the two previous years. All participant completed a nutritional questionnaire about their nutritional habits to ensure they were not taking any vitamins, minerals or other supplementation and to guarantee they all had a similar diet.

The athletes were classified into four groups: athletes group (SPG) (athletes; *n* = 80) of all the modalities (aerobic + anaerobic + aerobic-anaerobic) with an average age of 20.32 ± 3.24 years; aerobic athletes group (AEG) (Aerobic: long distance runners; *n* = 28) average aged of 21.58 ± 4.39 years; anaerobic athletes group (ANEG) (Anaerobic: judo and speed athletes; *n* = 24) with an average age of 20.11 ± 2.56 years; and aero-anaerobic athletes group (AE-ANEG) (Aerobic-anaerobic: professional football players; 28 individuals) with and average age of 22.23 ± 4.30. Sedentary students, with an average age of 21.78 ± 3.52 years, formed the control group (CG).

The beginning of the survey started at the end of October. At the moment of the beginning all athletes had started their respective training seasons. All SPG participants had been performing high level physical training, at least, two years before the beginning of the survey. AEG training routine consisted of 120 Km/week, performing 3–4 days/week in aerobic conditions or aerobic fartlek and 2–3 days/week of intense series and interval training. The training routine of the ANEG consisted of 5–6 training days/week, performing 3–4 h/day of specific routines. AE-ANEG participants started their competition in September. All AE-ANEG sportsmen performed an average of 3–4 h/day, 4–5 days/week of mixed physical (aerobic-anaerobic intensities), technical and tactical exercises.

#### Anthropometric measurements

The morphological characteristics of the participants were measured in the morning and always at the same time and in identical conditions. Body height was measured to the nearest 0.1 cm using a wallmounted stadiometer (Seca 220. Hamburg. Germany), and body weight was measured to the nearest 0.01 kg using calibrated electronic digital scales (Seca 769. Hamburg. Germany) in nude, barefoot conditions. Body fat content was estimated from sum of 4 skinfolds (abdominal, suprailiac, tricipital and subescapularis) and sum of 6 skinfolds (previous skinfolds, thigh and calf skinfolds). The ∑4 and ∑6 skinfold thicknesses were measured with a Harpenden calliper (Holtain Skinfold Caliper. Crosswell, UK). Measurements were made by the same operator, skilled in kinanthropometry techniques, in accordance with the International Society for the Advancement of Kinanthropometry recommendations. Heart rate and blood pressure were determined using an automatic sphygmomanometer (Omron HEM-780. Osaka. Japan) by a skilled technician, and after a five-minute rest period in supine position.

#### Physical performance evaluation

To evaluate the performance variables of the athletes and controls, an exercise test was performed in a treadmill (Powerjoc. Uk), equipped with a gas analyser (Metamax. Cortex Biophysik. Gmbh. Germany) and a Polar pulsometer (Polar. Norway). To ensure a warm-up phase, all participants ran before the test during 15 min ending at the initial speed of the test. For the test, the participants ran on a treadmill, incrementally in stages, until exhaustion, starting at a speed of 6 km·h-1 the CG and at 10 km·h-1 the SPG, increasing the speed at 1 km·h-1 every 400 m, with a stable slope of 1%, and always within the recommended parameters [9]. The two days before the test, training intensity was reduced, applying regenerative load in order to avoid pre-test fatigue.

The criteria used to determine maximal oxygen uptake (VO_2max_) was a stabilisation in oxygen uptake (VO_2_) accompanied of an increment in carbon dioxide (CO_2_) elimination, in respiratory exchange ratio (RER), and ventilatory volume (VE) induced by increments in exercise intensity (Km/h) in the treadmill. To ensure that physical tests were until exhaustion, athletes should come to the test in recovered conditions (normal basal parameters) and the RER had to exceed 1.

To verify the theoretical differences among sport groups and to ensure that each participant was properly classified in each group, and considering VO_2max_, maximal RER (RER_max_) was used. The justification of this criteria is based in the concept of RER, which defines de ratio between oxygen (O_2_) consumed and carbon dioxide (CO_2_) eliminated. When RER exceeds 1.0 it means that the metabolic production of CO_2_ overmatches the O_2_ metabolic utilization, which implies that the exceed in CO_2_ elimination comes from anaerobic metabolisms. This idea has been demonstrated by Goedecke et al. [10]. These authors demonstrated that RER is highly determined by lactate production. In this sense, anaerobic sportsmen can produce, buffer and tolerate more lactate than aerobic ones [11] which is reflected in higher RER_max_ values. Furthermore, aerobic sportsmen are highly affected by lactate accumulation, which supposes one of the main muscle fatigue precursors and one of the main performance limiting factors [12]. The inability of aerobic sportsmen to buffer and tolerate lactate is reflected in their RER_max_ values, which should be virtually lower than anaerobic sportsmen values.

#### Nutritional evaluation

In order to ensure participants were not taking any vitamins, minerals or other supplementations and in order to guarantee they were following a similar diet, all participants completed a dietary questionnaire. The questionnaire consisted in a 3-day daily nutritional record, in two pre-assigned weekdays and in one weekend day. In each day participants indicated, individually, the type, frequency and quantity (in grams) of every food consumed, then the nutritional composition of their diets was evaluated using different food tables and databases [13–15]. Results of this nutritional evaluation are collected in Tables 3 and 4.

#### Sample collection

Participants were recruited at 9 am for the extraction of 5 mL of venous blood from the antecubital vein, using a plastic syringe fitted with a stainless-steel needle. All extractions were carried out in fasting conditions. In order to obtain reliable results, all athletes were advised to rest the two previous days before the tests as well as not to train the morning before the extractions. The blood sample was collected into a metal-free polypropylene tube (previously washed with diluted nitric acid). Later, the blood sample was centrifuged at 2500 rpm for 10 min at room temperature to isolate the serum. The serum was aliquoted into an Eppendorf tube (previously washed with diluted nitric acid) and conserved at − 80 °C until the biochemical analysis.

### Experimental protocol

#### Serum trace elements determination

Cr, Cu, Mn, Ni and Se was performed by inductively coupled plasma mass spectrometry (ICP-MS). Decomposition of the organic matrix was performed by heating for 10 h at 90 °C after the addition of 0.8 mL HNO_3_ and 0.4 mL H_2_O_2_ to 1 mL of serum [16]. The samples were then dried at 200 °C on a hot plate. Sample reconstitution was carried out by adding 0.5 mL of nitric acid, 10 μL of In (10 mg·L-1) as internal standard and ultrapure water to complete 10 mL. Reagent blanks, element standards and certified reference material (Seronorm, lot 0511545, Sero AS Billingstand, Norway) were prepared in the same way and used for accuracy testing. Before the analysis, the commercial control materials were diluted according to the recommendation of the manufacturer. Digested solutions were assayed by an ICP-MS Nexion model 300D (PerkinElmer, Inc., Shelton, CT, USA) equipped with a triple quadrupole mass detector in addition to a reaction cell/collision that allows operation in three modes: STD (no reaction gas), KED or kinetic energy discrimination (with helium as the collision gas) and DRC or reaction mode (with ammonia as the reaction gas). Both collision and reaction gases such as argon plasma had a purity of 99.999% and were supplied by Praxair (Madrid, Spain). Two mass flow controllers regulated gas flows. The frequency generator was free-swinging and worked at 40 Mhz. Three replicates were analysed per sample. Quantification was performed by indium (In) as internal standard. The values of the standard materials of each element (10 μg·L-1) measured for quality controls were in good agreement with intra and inter-assay variation coefficients of less than 5%.

#### Statistical evaluations

Statistical analyses were performed with the *IBM SPSS Statistic* software version 19 for *Windows*. The results are expressed as *x* ± *s*, where *x* are the mean values and *s* the standard deviation. A *p*-value of p ≤0.05 was considered statistically significant.

The normal distribution of the variables was assessed using the Shapiro-Wilks test. Student T test was used to evaluate differences between CG and SPG, and One-way Anova test followed by Bonferroni post hoc test were used to determine differences between CG, AEG, ANEG and AE-ANEG. The differences were evaluated in the anthropometric, cardiorespiratory, nutritional and seric variables.

## Results

### Anthropometric and cardiorespiratory characteristics

Table 1 shows data obtained in the anthropometric and cardiorespiratory evaluations from the two groups. It can be observed that SPG had a total body weight and fat values (Σ of skinfolds) significantly lower (*p* < 0.001) than the CG. VO_2max_ was significantly higher (*p* < 0.001) in athletes compared to sedentary group. Resting heart rate was lower (*p* < 0.01) in athletes in respect to the control group. No differences were found in the age of all participants. As in can be observed in this survey the different groups are well-characterized.

**Table 1:** Anthropometric characteristics of control group and athletes

	CG	SPG
	(*n* = 31)	(*n* = 80)
Height (m)	1.76 ± 0.057	1.76 ± 0.07
Weight (kg)	78.21 ± 12.19	65.31 ± 7.55***
Age (years)	21.78 ± 3.52	20.32 ± 3.24
Σ4 skinfolds (mm)	52.02 ± 23.77	35.12 ± 9.29***
Σ6 skinfolds (mm)	88.82 ± 34.50	45.85 ± 16.69***
VO_2Máx_ (mL·min-1·kg-1)	41.89 ± 7.50	64.08 ± 5.27***
HR _basal_ (beat·min-1)	71.58 ± 7.38	59.75 ± 9.66*

Student test (*p < 0.05, **p < 0.01; ***p < 0.001) differences between control and sportsmen groups

Table 2 shows data about the SPG, separated by metabolic exercise specialty. AEG and ANEG show lower (*p* < 0.001) body weight, followed by AE-ANEG (*p* < 0.05). However, in relation to fat, AEG have the least (*p* < 0.001) values followed by ANEG (*p* < 0.001) and AE-ANEG(*p* < 0.01). In relation to the oxygen update, the AEG have a higher (*p* < 0.001) value followed by ANEG (*p* < 0.001) and AE-ANEG (*p* < 0.01) in respect the CG. For its part, resting heart rate was lower (*p* < 0.01) in AEG followed by AE-ANEG (*p* < 0.05) and ANEG (*p* < 0.05). Furthermore, it was found significant differences in the RER_max_ of AEG and ANEG in comparison to CG, presenting the first group the lowest (*p* < 0.05) values and the second one the highest (*p* < 0.05) results.

**Table 2:** Characteristics of control group and sportsmen classified by metabolic modalities

	CG	AEG	ANEG	AE-ANEG
	(*n* = 31)	(*n* = 28)	(*n* = 24)	(*n* = 28)
Height (m)	1.76 ± 0.05	1.77 ± 0.05	1.73 ± 0.07	1.80 ± 0.05
Weight (kg)	78.21 ± 12.19	64.95 ± 7.10***	64.91 ± 8.46***	73.78 ± 6.12*^†††■■■^
Age (years)	21.78 ± 3.52	21.58 ± 4.39	20.11 ± 2.56	22.23 ± 4.30
Σ4 skinfolds (mm)	52.02 ± 23.77	32.56 ± 8.75***	33.66 ± 9.87***	38.25 ± 10.06**^†^
Σ6 skinfolds (mm)	88.82 ± 34.50	49.69 ± 14.84***	56.32 ± 16.65**^††^	59.49 ± 17.10**^†††^
VO_2Máx_ (mL·min^− 1^·kg^− 1^)	41.89 ± 7.50	66.17 ± 8.36***	61.23 ± 3.00***^†^	59.85 ± 4.53**^††■^
RER_max_	1.17 ± 0.10	1.10 ± 0.08*	1.25 ± 0.06*	1.15 ± 0.09
HR_basal_ (beats·min^− 1^)	72.58 ± 7.38	54.90 ± 10.85**	62.85 ± 7.57*^††^	57.21 ± 10.51*^■^
HRmax (beats·min^− 1^)	184.32 ± 12.71	173.44 ± 8.3*	195.10 ± 8.45^■^	174.62 ± 11.68

Anova and Bonferroni. (* *p* < 0.05; ** *p* < 0.01; *** *p* < 0.001) differences between control group and aerobic, anaerobic and aerobic-anaerobic groups; († *p* < 0.05; †† *p* < 0.01; ††† *p* < 0.001) differences between aerobic group and anaerobic and aerobic-anaerobic groups; (■ *p* < 0.05; ■■ *p* < 0.01; **■■■***p* < 0.001) differences between anaerobic group and aerobic-anaerobic group

### Nutritional intake of minerals

Table 3 collects data about the nutritional intake of all participants. It can be observed in this table that sportsmen presented higher (*p* < 0.01) caloric intake than controls. No significant differences were found among groups in the intake of any mineral.

**Table 3:** Nutritional intake of trace elements with essential functions demonstrated: Cr, Cu, Mn, Ni and Se in control group and sportsmen

Elements	CG	SPG
(Reference Daily Intake)	(*n* = 31)	(*n* = 80)
Energy (Kcal/d)	2243.64 ± 251.98	2860 ± 198.31**
Cr (50–200 μg/d)	95.71 ± 53.30	125.71 ± 63.53
Cu (2000–3000 μg/d)	1842.10 ± 636.21	1715.00 ± 615.01
Mn (2500–5000 μg/d)	3053.66 ± 1222.17	3216.80 ± 1360.18
Ni (50–150 μg/d)	102.25 ± 15.73	108.66 ± 16.62
Se (50–200 μg/d)	100.82 ± 18.24	97.31 ± 19.48

Student test (**p < 0.01) differences between control and sportsmen groups

Table 4 presents data about the nutritional intake of the subjects of the different groups. It can be observed that only AG presented higher (*p* < 0.001) caloric intake than CG.

**Table 4:** Nutritional intake of trace elements Cr, Cu, Mn, Ni and Se of control group and sportsmen separated by metabolic specialties

Element	CG	AEG	ANEG	AE-ANEG
	(*n* = 31)	(*n* = 28)	(*n* = 24)	(*n* = 28)
Energy (Kcal/d)	2243.64 ± 251.98	2989.12 ± 200.22***	2215.22 ± 265.15	2571.13 ± 211.56
Cr (μg/d)	95.71 ± 53.30	145.71 ± 74.13	110.42 ± 73.10	94.71 ± 53.30
Cu (μg/d)	1842.10 ± 636.21	1642.00 ± 761.01	1874.75 ± 687.14	1714.23 ± 742.31
Mn (μg/d)	3053.66 ± 1222.17	3367.22 ± 1432.12	3200.24 ± 1422.21	2998.28 ± 1547.12
Ni (μg/d)	102.25 ± 15.73	110.31 ± 17.44	99.22 ± 16.93	104.72 ± 17.33
Se (μg/d)	100.82 ± 18.24	97.31 ± 19.48	99.17 ± 28.15	89.01 ± 24,11

Anova and Bonferroni tests. *** *p* < 0.001

### Serum mineral concentrations

Table 5 shows serum essential trace minerals data of CG and SPG. It is interesting that in the SPG exist higher basal concentrations of Cr, Cu, Mn and Ni, being statistically significant in the cases of Cr, Ni (*p* < 0,001) and Mn (*p* < 0.05). Aversely, basal serum Se concentrations of SPG were lower than CG; being this diminution highly significant (*p* < 0.001).

**Table 5:** Serum trace element concentrations of control group and high-level sportsmen

Element	CG	SPG
	(*n* = 31)	(*n* = 80)
Cr (μg·L^− 1^)	0.22 ± 0.24	1.92 ± 3.13***
Cu (μg·L^− 1^)	759.46 ± 108.40	784.63 ± 143.03
Mn (μg·L^− 1^)	1.185 ± 0.44	1.63 ± 1.46*
Ni (μg·L^− 1^)	0.51 ± 0.41	1.93 ± 2.22***
Se (μg·L^− 1^)	102.37 ± 12.99	85.64 ± 17.86***

Student test (*p < 0.05, **p < 0.01; ***p < 0.001) differences between control group and sportsmen group

Table 6 shows the serum concentrations of CG and SPG separated by metabolic modalities. Cr presents higher (*p* < 0,001) levels in all sport modalities than in CG, being the highest concentration of AEG, followed by AE-ANEG (*p* < 0.001) and ANEG (*p* < 0.001).

**Table 6:** Serum trace elements concentrations of control group and sportsmen separated by metabolic specialties

Element	CG	AEG	ANEG	AE-ANEG
	(*n* = 31)	(*n* = 28)	(*n* = 24)	(*n* = 28)
Cr (μg·L^−1^)	0.15 ± 0.20	1.43 ± 4.10***	0.44 ± 0.24***^†††^	0.70 ± 0.504***^†††■^
Cu (μg·L^− 1^)	750.46 ± 113.47	741.49 ± 114.15	767.66 ± 74.83	857.91 ± 180.55**^††■^
Mn (μg·L^− 1^)	1.10 ± 0.41	2.54 ± 1.74***	0.81 ± 0.24**^†††^	1.02 ± 0.39***^†††■^
Ni (μg·L^−1^)	0,47 ± 0.43	2.87 ± 2.95***	1.11 ± 0.35***^††^	1.00 ± 0.69**^††^
Se (μg·L^− 1^)	102.87 ± 13.80	94.16 ± 13.61*	79.17 ± 11.48***^†††^	84.01 ± 23.43*^†^

Anova and Bonferroni tests. (*p < 0.05; **p < 0.01; ***p < 0.001) differences between control group and aerobic, anaerobic and aerobic-anaerobic groups; (†*p* < 0.05; ††*p* < 0.01; †††*p* < 0.001) differences between aerobic group and anaerobic and aerobic-anaerobic groups; (■*p* < 0.05; ■■*p* < 0.01; **■■■***p* < 0.001) differences between anaerobic group and aerobic-anaerobic group

In relation to Cu, it can be observed that only AE and AENG have similar concentrations than CG. Only serum concentrations of Cu in AE-ANEG participants were higher (*p* < 0,01) than CG. In comparison with the other exercise modalities this sportsman, also, has higher (*p* < 0,01) concentration than AEG and ANEG (*p* < 0,05).

In the analysis of Mn, separated by exercise specialties, important changes can be found. Only AEG present higher (*p* < 0,001) concentrations than CG. Contrary, the other sport modalities present different Mn concentrations; both groups shown lower concentrations than CG, being the most significance (*p* < 0,001) the AE-ANEG group followed by ANEG (*p* < 0,01). Therefore, the highest concentrations are present in AEG, followed by CG, AE-ANEG and AEG.

Ni presented higher (*p* < 0,001) concentrations in all sportsmen in respect to control group. However, Ni highest concentrations were found in AEG (*p* < 0,001), followed by ANEG (*p* < 0,001) and AE-ANEG present the lowest (*p* < 0,01) concentrations.

Se presented lower serum concentrations in all sportsmen respect CG (*p* < 0,001). The lowest (*p* < 0,001) concentrations were founded in ANEG group, followed by AE-ANEG group (*p* < 0,01). The highest (*p* < 0,05) Se concentrations belonged to AEG.

## Discussion

One of the main exposure sources to trace elements is diet [13]. In this survey, nutritional intake was analysed by recording each gram of each food ingested during the evaluation days. Once the total daily amounts of each aliments were summed, the amount of each specific mineral was obtained using several databases [13–15].

In this survey participated sedentary population (CG) as well as high level sportsmen of different modalities (Aerobic, Anaerobic and Mixed). A critical issue in this kind of surveys is a proper delineation of experimental groups in accordance with the predominant metabolic demands (aerobic, anaerobic or aerobic-anaerobic metabolisms), because each specific sport induces different biological adaptations according to the metabolisms developed in of each activity. RER_max_ could be an interesting variable to differentiate groups due to the link between RER values and anaerobic CO_2_ production [10]. Data about RERs in Table 2 manifests proper differences among sports groups and was used as criteria to verify that sportsmen groups was accurately delineated.

Sportsmen have, in comparison to sedentary population, greater energetic demands, due to their physical training. This fact is reflected in the obtained results, being the caloric intake (Kcal/d) significantly higher (*p* < 0.01) among sportsmen, but only in the AEG. Theoretically, sportsmen should present, due to an augmented food intake, greater mineral intake, but, contradictorily, the obtained results have shown similar mineral values. This fact was previously analysed in a review of Gupta and Gupta [17]. In this review, it was reported that although takin a balanced and varied diet there is a great percentage of possibilities to have a lack of some elements. The main reason of this fact is the chemical composition of grounds, poor in several minerals. This idea can explain why the sportsmen of this survey have similar nutritional intake of minerals in spite of ingest sensibly higher amounts of foods. Another possible explanation could be that athletes ingested low amounts of foods rich in the studied elements.

Despite similar nutritional intake of minerals. it can be observed significant differences in serum concentrations of essential trace metals between sportsmen and sedentary people. However, mineral seric values were within reference concentrations [18] with the exception of Cr, which presented concentrations under normal values in the CG. This fact could be due to a diminution in the intake, as Clarkson [19] indicated, fact that has not been reflected in the obtained results.

Seric Cr concentrations were higher (*p* < 0.001) in SPG in comparison to CG. Berger et al. [20], found in marathon runner’s values of this element similar to the results obtained in this survey. However, they had not control group. They also reported high Cr concentrations before and after the race, being sensibly higher than in our sportsmen. Normal or sensibly augmented Cr values could be highly relevant to sportsmen because of the link of this element with insulin, a critical hormone for high level physical demands [1–3].

If sportsmen are classified by metabolic modalities and compared to the CG it can be observed that all sportsmen, independently of the nature of the exercise, presented greater Cr values than controls. The highest Cr values are reported in the AEG, followed by the AE-ANEG, and being the lowest in the ANEG. This fact could be linked to the insulin-related Cr role, being critical in aerobic modalities, where insulin regulates the substrates metabolism; in the other two modalities, this hormone takes a less important role. It is well known that the practice of physical exercise decreases the blood glucose and insulin levels. These effects may be observed even in only one exercise session and may last for several hours after the end of the effort. If regular practice of physical exercise is performed, a chronic blood glucose diminution may occur [21].

Long-term Cr supplementation effects have been studied involving long periods of time (12–24 weeks). It has been associated high daily doses of chromium (400 μg·day^− 1^) with significant reductions in body fat mass of 5% [22–25]. This fact indicates that the effects of chromium on body re-composition may occur; however, these effects require high dosage, long supplementation, periods.

In the present survey, the highest Cr serum concentrations have been found in AEG, which is the group with the lowest body fat amounts. This fact can be mainly due to physical exercise by itself but, in all cases, is in accordance with the previous reports and it could strengthen the hypothesis of Rubin et al. [26], who indicated that body composition changes, body fat diminutions or free fat weight increments can be produced by physical exercise by itself or by the exercise-associated serum Cr increments.

In the case of Cu, no differences were found between the SPG and the CG. However, if SPG participants are separated by metabolic modalities, serum Cu concentrations are significantly higher (*p* < 0.01) in the AE-ANEG in comparison to the CG and higher in the ANEG than in the AEG (*p* < 0.01). Cu has been previously studied in plasma of high level aerobic and anaerobic sportsmen compared to a control group (who performed moderate physical activities) [27]. The results of this previous research showed that anaerobic sportsmen had significantly higher concentrations than controls and aerobic sportsmen. The results obtained here are in accordance to this previous report, being the highest concentrations in football players who performed aerobic-anaerobic training programs. The Cu concentrations in the AE-ANEG concentrations were also higher (*p* < 0.01) than in the CG. The increase on these concentrations could be due to exercise-induced rhabdomyolysis, as consequence of daily training. When muscle tissues impact increases, as occur in high-intensity activities, this effect is augmented [28].

It could be stated that aerobic exercises develop more reductions in blood Cu levels among athletes [29]. Resina, Gatteschi, Rubenni, Giamberardino, and Imreh [30] found among soccer players lower serum ceruloplasmin levels and lower serum ceruloplasmin biological activity than in the control group. However, serum Cu levels were comparable in both groups. These results suggest that more attention should be focussed to serum Cu and ceruloplasmin among soccer players.

Mn is a compound of superoxide dismutase enzyme (Mn-SOD). Thereby, in several researches, it has been observed that physical exercise increases the Mn-SOD activity in the myocardium and in the mitochondria [31–33]. This is a critical fact among sportsmen because Mn-SOD is a mitochondrial antioxidant enzyme which neutralize superoxide radicals formed during the exercise. Furthermore, Mn develops important functions in gluconeogenesis and urea formation processes as an active compound of several involved enzymes. These processes are essential, becoming critical in aerobic sportsmen [31, 33]. Sánchez, López-Jurado, Aranda, and Llopis [34] in a work developed in the south of Spain, studied among sedentary and physically active populations Mn blood concentrations. These authors found the highest concentrations in active participants. The higher Mn concentrations in sportsmen would be linked to an increased organic utilization of this mineral in the mitochondrial Mn-SOD production processes, among others [34]. Higher basal blood Mn concentrations in aerobic athletes in comparison with other metabolic sportsmen could be linked to a probably lower iron (Fe) levels, fact that is common in long distance sportsmen, as a consequence of the impact of Fe body levels in Mn metabolism [35, 36]. In this sense, it has been observed increased blood Mn levels in Fe deficitary cases [37, 38]. The fact that the AEG had the higher concentrations, followed with the AE-ANEG and the ANEG in our study, verifies and reinforces the previous reports.

Ni controls tissular levels of phospholipids, triglycerides, urea, glucose, glycogen and ATP [4, 5]. Due to these roles is critical among sportsmen to maintain high organic concentrations of this mineral. Berger et al. [20] found in marathon runners similar blood Ni levels that in the present survey. However, they had no control group.

The highest (*p* < 0.001) Ni concentrations in this survey have been reported in sportsmen. This fact could be linked to their high training volume as well as to the higher daily quantities of metabolic reactions needed to maintain high performance levels, specially among aerobic sportsmen. As previously indicated, it is known that Ni regulates tissular concentrations of substrates and metabolites, which are significantly altered during long-duration trainings and competitions. The obtained data reinforces this idea, being in aerobic participants the highest concentrations, followed by anaerobic and aerobic-anaerobic exercises (*p* < 0.001).

About the role of Se in sportsmen, it is known that physical activity generates an increase in free radicals of oxygen production, being this the fact which conduces sportsmen to improve their antioxidant system, as adaptive mechanism. A specific adaptive change is the behaviour of the enzyme GSH-Px, a Se protein [39].

The obtained results manifest lower Se concentrations in all sportsmen in comparison to CG. Sánchez et al. [34] also found lower Se values in active population in comparison to sedentary people. Se intake from food was not enough to maintain the constant levels of blood Se during training. Because the estimated intake of 48 μg·day-1 was nevertheless higher than the lower recommended level (30 μg·day-1), it should be considered that in sportsmen population the recommendation should be increased [40]. The main reason of this affirmation is that Se requirements are increased among athletes [41].

This could be an important cause of the low Se concentrations found in our sportsmen. An augmented production of GSH-Px and a major synthesis of other Se-proteins could be a reasonably justification of the lower Se values among sportsmen.

In the present study the lowest concentration was found in the ANEG (*p* < 0.001) followed by the AE-ANEG and the AEG. Pograjc et al. [40] found decreased Se plasma levels during training of intensive physical activity. This fact suggests a slowly biologically active decreasing Se pool. The results of the study of Akil et al. [39] indicated that decreased liver glycogen levels in acute swimming exercise can be restored by selenium administration. It is possible, so, that the cause of the lowest Se concentrations in the ANEG and the AE-ANEG was a greater and more rapid replacement of muscular glycogen necessary for a high performance in these modalities. The results of the study of Akil et al. [39] indicate that the increase in free radical production and lactate levels due to acute swimming exercise in rats might be offset by selenium supplementation. Selenium supplementation may be important in that it supports the antioxidant system in physical activity. Physical activities which produce lactate and high amounts of free radicals are anaerobic, so, as the previous work indicated, anaerobic sportsmen would need more Se quantities to overcome the exercise-induced metabolic situations as well as to avoid oxidative damage. This could explain the obtained data.

A nutritional plan rich in Se containing foods may be beneficial for both athletes who exercise regularly and in patients with increasing oxidative stress [42].

Finally, regarding the limitations of this survey it has to be mentioned that to know the exact metabolic behaviour of each element, more matrixes have to be analysed, like urine, plasma, or sweat. The inclusion of these other matrixes could inform about the degree of elimination and redistribution of each element. In further surveys, multi-matrix studies should be carried out tin order to deep in the knowledge of the exact effect of diet and exercise in the organic trace elements values.

## Conclusions

This research manifests that essential mineral elements play a critical role in the metabolism of continuous physical practice cases. As consequence of specific physical training, sportsmen serum concentrations suffer different changes related to the nature of the efforts and training modality practised.

In some cases, the upper demands induced by high-level training make necessary to maintain high serum bioavailability of these elements to ensure quick responses to the exercise-induced cellular changes as well as to prevent considerable losses which could reduce physical performance.

So, these findings should be considered to develop specific supplementation processes, when it be necessary. This plan seems critically important in the case of Se. Also, research/evaluations of this elements are essential for trainers and high-level sportsmen, to prevent performance injuries during the season and to ensure an optimum physiologic status.
